# Supplementary material for: Glycidamide Promotes the Growth and Migratory Ability of Prostate Cancer Cells by Changing the Protein Expression of Cell Cycle Regulators and Epithelial-to-Mesenchymal Transition (EMT)-Associated Proteins with Prognostic Relevance
Source: Int J Mol Sci. 2019 May 4;20(9):2199. doi: 10.3390/ijms20092199 (PMC6540322; doi:10.3390/ijms20092199)
Supplement: Supplementary file 1 [file ijms-20-02199-s001.pdf]

Glycidamide Promotes the Growth and Migratory Ability of Prostate Cancer Cells by Changing the Protein Expression of Cell Cycle Regulators and Epithelial-to-Mesenchymal Transition (EMT)-Associated Proteins with Prognostic Relevance

Titus I. Ekanem[1,2], Chi-Chen Huang[3,†], Ming-Heng Wu[4,†], Ding-Yen Lin[5,6], Wen-Fu T. Lai[7,8,9] and Kuen-Haur Lee[6,10,*]

[1]Ph.D. Program for Cancer Molecular Biology and Drug Discovery, College of Medical Science and Technology, Taipei Medical University and Academia Sinica
[2]Department of Hematology, University of Uyo,Uyo, Nigeria
[3]Graduate Institute of Neural Regenerative Medicine, College of Medical Science andTechnology, Taipei Medical University, Taipei, Taiwan
[4]Graduate Institute of Translational Medicine, Taipei Medical University, Taipei, Taiwan.
[5]Department of Biotechnology and Bioindustry Sciences, College of Bioscience and Biotechnology, National Cheng Kung University, Tainan
[6]Graduate Institute of Cancer Biology and Drug Discovery, College of Medical Science and Technology, Taipei Medical University, Taipei, Taiwan
[7]McLean Imaging Center, McLean Hospital, Harvard Medical School, Belmont, MA, United States of America
[8]Department of Research, Taipei Medical University/Shuang-Ho Hospital, New Taipei City, Taiwan
[9]Department of Dentistry, Taipei Medical University/Shuang-Ho Hospital, New Taipei City, Taiwan
[10]Ph.D. Program for Cancer Molecular Biology and Drug Discovery, College of Medical Science and Technology, Taipei Medical University, Taipei, Taiwan

[†]These authors contributed equally to this work.

* Correspondence: Dr. Kuen-Haur Lee, Graduate Institute of Cancer Biology and Drug Discovery, College of Medical Science and Technology, Taipei Medical University, No. 250 Wu-Hsing Street, Taipei 11031, Taiwan. Tel: +886-2-27361661 ext. 7627, Fax: +886-2-66387537, E-mail: khlee@tmu.edu.tw

Supplementary Material

Figure S1. Survival analysis of prostate cancer patients with SurvExpress (n=140). Low expression of CCND1 (**A**), CDK4 (**B**), and TWIST1 (**C**) were correlated with good prognosis of prostate cancer patients. High expression of SNAI1 (**D**), SNAI2 (**E**), and CDH1 (**F**) were correlated with good prognosis of prostate cancer patients.

Figure S2. Kaplan–Meier curves according to any two-gene models. Clinical outcomes for the combinations of CDK4/TWIST1 (**A**), CDK4/SNAI2 (**B**), and TWIST1/SNAI2 (**C**) mRNA status of prostate cancer patients.

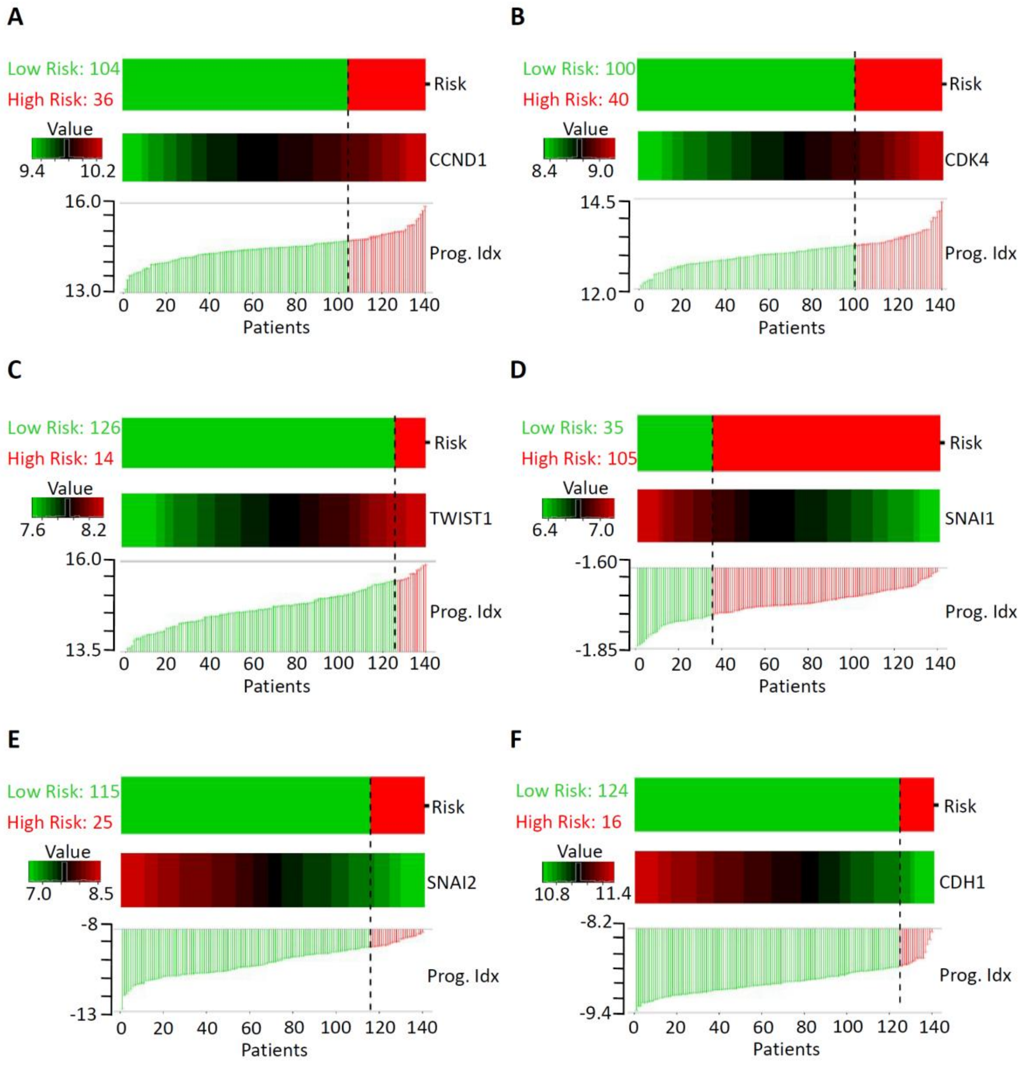

Supplementary Fig. 1

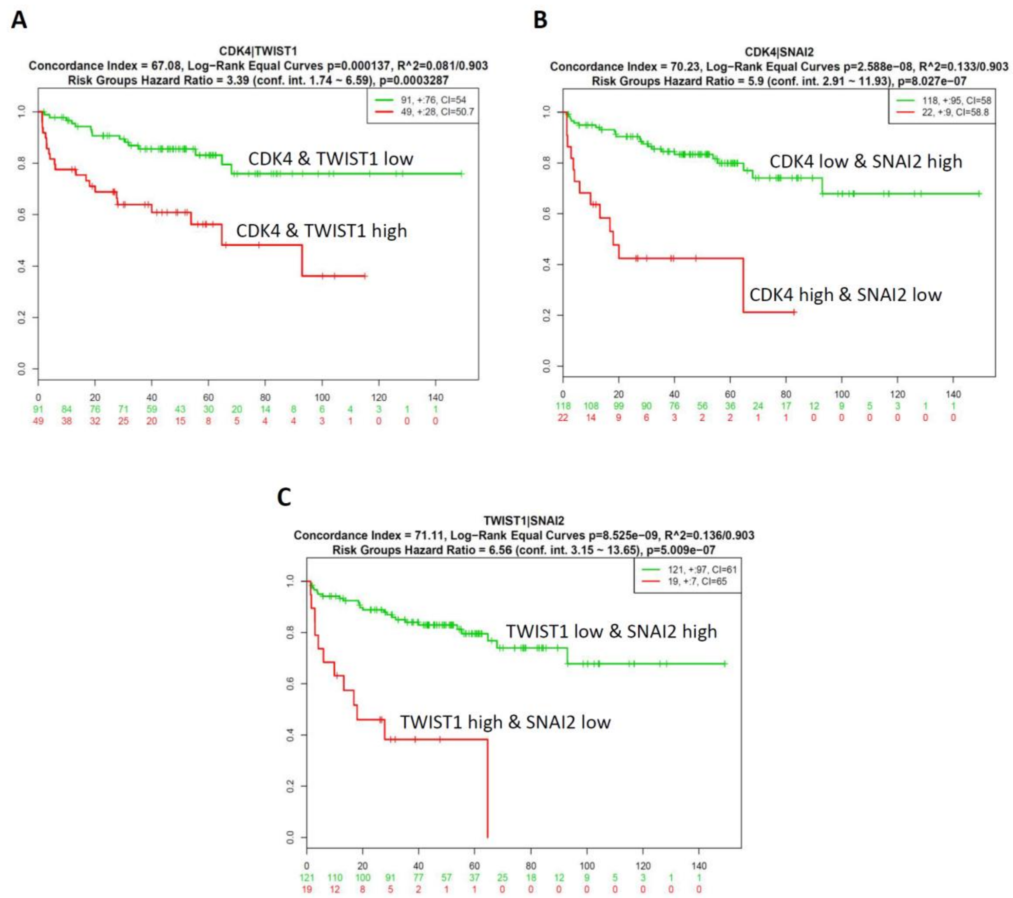

Supplementary Fig. 2
